# Bis[2-(2-hy­droxy­meth­yl)pyridine-κ^2^ *N*,*O*](pivalato-κ*O*)copper(II)

**DOI:** 10.1107/S1600536812030917

**Published:** 2012-07-14

**Authors:** M. Mobin Shaikh, Veenu Mishra, Priti Ram, Anil Birla

**Affiliations:** a Discipline of Chemistry, School of Basic Sciences, IIT Indore, Indore, Madhya Pradesh 452 017, India

## Abstract

The structure of the centrosymmetric title complex, [Cu(C_5_H_9_O_2_)_2_(C_6_H_7_NO)_2_], has the Cu^II^ atom on a centre of inversion. The Cu^II^ atom is six-coordinate with a distorted octa­hedral geometry, defined by the N and O atoms of the chelating 2-(2-hydroxymethyl)pyridine ligands and two carboxyl­ate O atoms from two monodentate pivalate ions. The crystal packing is stabilized by inter­molecular C—H⋯O and intra­molecular O—H⋯O hydrogen-bond inter­actions.

## Related literature

For pyridine alcohol-based biomimetic sensors, see: Shaikh *et al.* (2010 ▶). For solid-state transformations, see: Shaikh *et al.* (2009 ▶, 2010 ▶). For structures with pyridine alcohols, see: Hamamci *et al.* (2004 ▶); Lah *et al.* (2006 ▶).
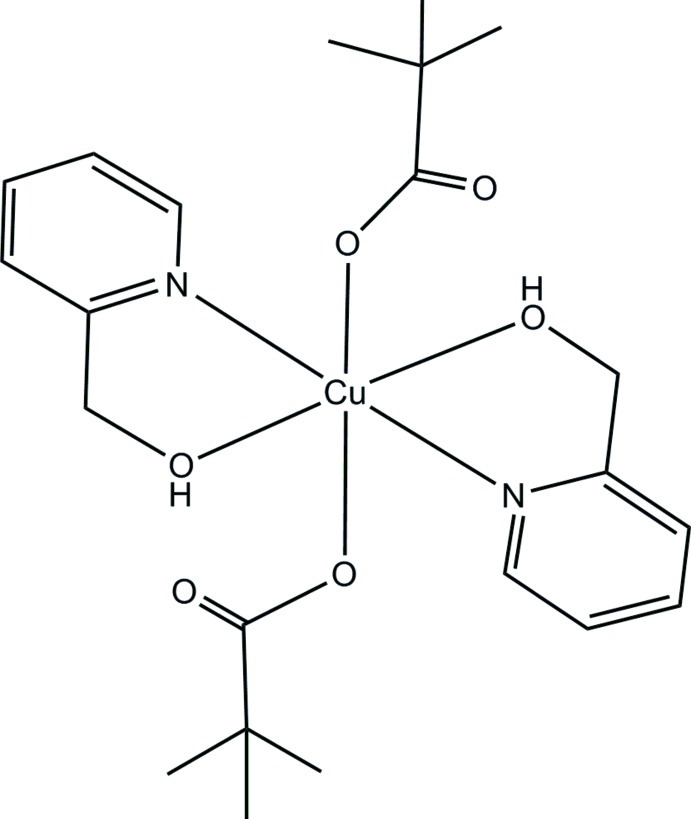


## Experimental

### 

#### Crystal data

[Cu(C_5_H_9_O_2_)_2_(C_6_H_7_NO)_2_]
*M* *_r_* = 484.04
Monoclinic, 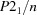

*a* = 9.797 (5) Å
*b* = 8.829 (5) Å
*c* = 13.674 (5) Å
β = 91.907 (5)°
*V* = 1182.1 (10) Å^3^
*Z* = 2
Cu *K*α radiation
μ = 1.63 mm^−1^
*T* = 150 K
0.33 × 0.28 × 0.23 mm

#### Data collection

Oxford Super Nova diffractometer
Absorption correction: multi-scan (*CrysAlis RED*; Oxford Diffraction, 2009 ▶) *T*_min_ = 0.615, *T*_max_ = 0.706
6929 measured reflections
2282 independent reflections
2052 reflections with *I* > 2σ(*I*)
*R*_int_ = 0.036

#### Refinement

*R*[*F* ^2^ > 2σ(*F* ^2^)] = 0.040
*wR*(*F* ^2^) = 0.119
*S* = 1.06
2282 reflections
146 parameters
H atoms treated by a mixture of independent and constrained refinement
Δρ_max_ = 0.43 e Å^−3^
Δρ_min_ = −0.54 e Å^−3^

### 

Data collection: *CrysAlis CCD* (Oxford Diffraction, 2009 ▶); cell refinement: *CrysAlis CCD*; data reduction: *CrysAlis RED* (Oxford Diffraction, 2009 ▶); program(s) used to solve structure: *SHELXS97* (Sheldrick, 2008 ▶); program(s) used to refine structure: *SHELXL97* (Sheldrick, 2008 ▶); molecular graphics: *DIAMOND* (Brandenburg, 1999 ▶); software used to prepare material for publication: *publCIF* (Westrip, 2010 ▶).

## Supplementary Material

Crystal structure: contains datablock(s) I, global. DOI: 10.1107/S1600536812030917/bt5967sup1.cif

Structure factors: contains datablock(s) I. DOI: 10.1107/S1600536812030917/bt5967Isup2.hkl

Additional supplementary materials:  crystallographic information; 3D view; checkCIF report

## Figures and Tables

**Table 1:** Hydrogen-bond geometry (Å, °)

*D*—H⋯*A*	*D*—H	H⋯*A*	*D*⋯*A*	*D*—H⋯*A*
O1—H101⋯O3	0.95 (4)	1.64 (4)	2.588 (2)	171 (3)
C2—H2⋯O3^i^	0.95	2.57	3.289 (3)	132
C4—H4⋯O3^ii^	0.95	2.50	3.392 (3)	157

Symmetry codes: (i) 
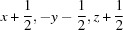
; (ii) 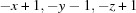.

**Fig. 1.**
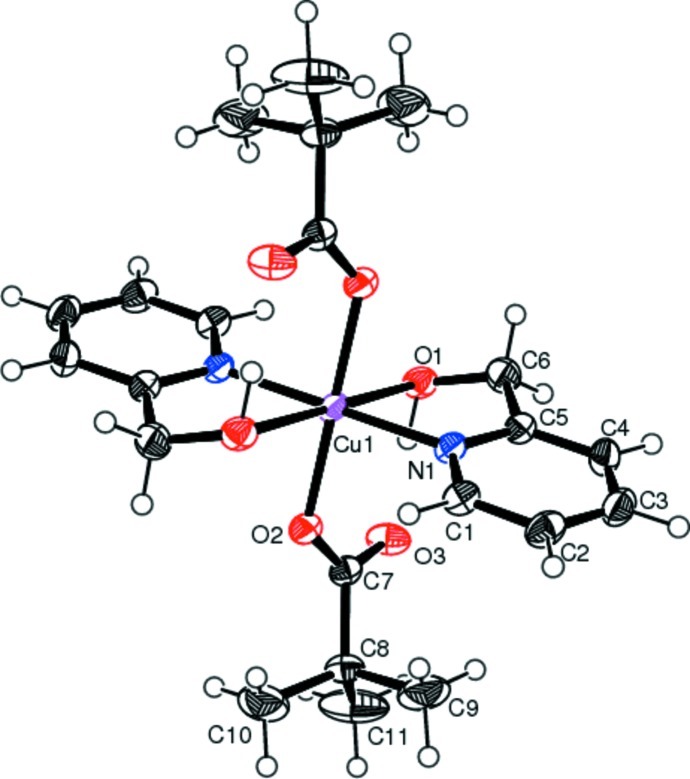
View of the molecular structure of the title compound with displacement ellipsoids drawn at the 50% probability level. The symmetry-related moiety was generated by (-x, -y, -z).

**Fig. 2.**
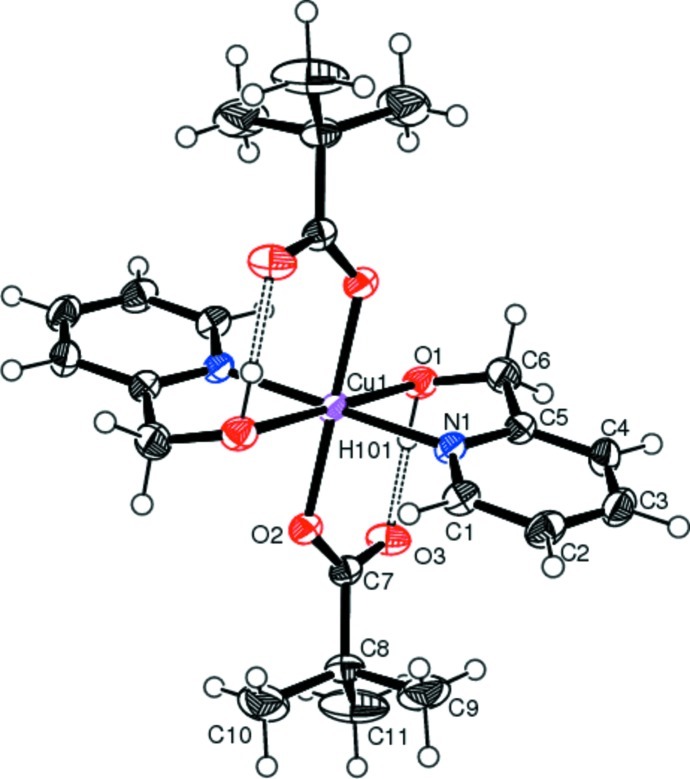
Intra-molecular hydrogen bonding in the title compound.

**Fig. 3.**
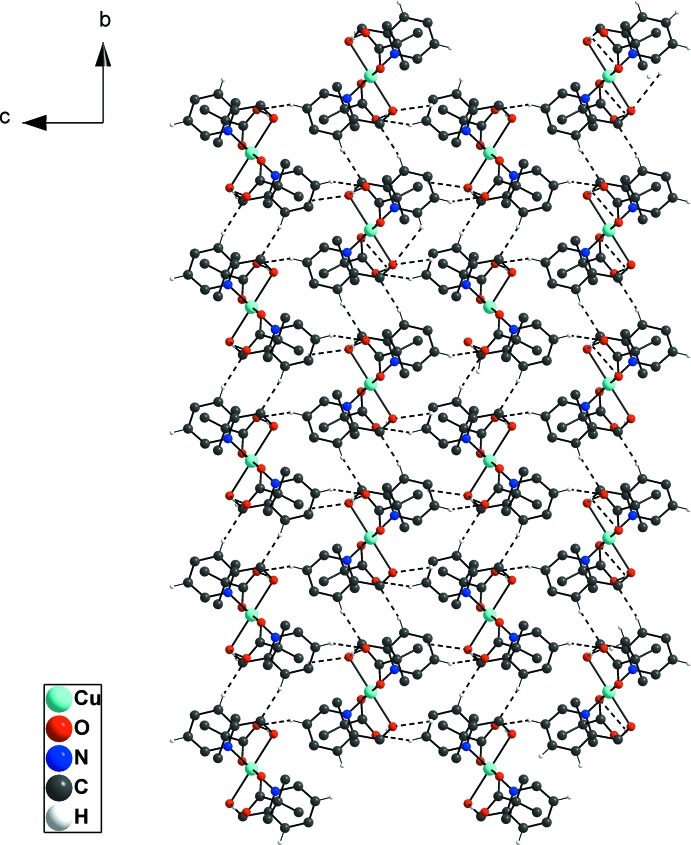
A perspective view of the hydrogen bonded two-dimensional-network; view along the a-axis. Hydrogen bonds are drawn as dashed lines.

## supplementary crystallographic information

### Comment

We have reported a series of pyridine alcohol based Cu(II) complexes with a range of applications such as biomimetic sensors (Shaikh *et al.*, 2010) and solid-state transformations (Shaikh *et al.*, 2009). Pyridine alcohols are used because they possess two functional groups, both having the ability to bind the metal centres (Hamamci *et al.*, 2004; Lah *et al.*, 2006).

Herein we report synthesis and crystal structure of a mononuclear Cu(II) complex with hmp-H acting as a bidentate chelating ligand. The Cu(II) atom is surrounded by two N and O atoms from hmp-H in a basal plane and the apical positions are occupied by two O atoms from monodentate pivalate group forming a distorted octahedral geometry (Fig. 1).

The packing reveals intra (O—H···O) and inter (C—H···O) hydrogen bonds. The intramolecular hydrogen bonding involves the alcoholic OH group of hmp-H and an O atom of the pivalate group (Fig. 2). The intermolecular C(4)—H(4)···.O(3) hydrogen bond involves an H-atom of pyridine ring and an O atom of the pivalate group forming one-dimensional chain along the b-axis which binds to a neighbouring one-dimensional chain *via* C(2)—H(2)···O(3) along *c*-axis, leading to the formation of hydrogen bonded two-dimensional network (Fig. 3).

### Experimental

A solution of pivalic acid (102 mg, 1.0 mmol) in 10 ml methanol was added to a 30 ml methanolic solution of Cu(CH_3_COO)_2_.2H_2_O (199 mg, 1.0 mmol) and hmp-H (109 mg, 1.0 mmol). The resultant solution was stirred for 12 h at room temperature. The solution was then passed through filter paper (Whatman filter paper, 70 mm) in order to remove any unreacted materials. The filtrate was allowed to stand at room temperature for crystallization. On slow evaporation light-blue single crystals of [Cu(C_5_H_7_ON)_2_(C_5_H_9_O_2_)_2_] were obtained after 2–3 d. M.p. 476–478 K. Yield: 88%. Anal. Calcd for C_22_H_32_CuN_2_O_6_ (Mr = 484.04): C, 54.59; H, 6.66; N, 5.79. Found: C 54.62; H 6.70; N 5.76.

### Refinement

H atoms bonded to C were placed geometrically and treated as riding on their parent atoms, with C—H 0.95 (pyridyl), C—H 0.99 (methylene) Å [*U*_iso_(H) = 1.2 *U*_eq_(C) or 1.5 *U*_eq_(C_methyl_)]. The hydroxyl H atom was freely refined.

### Crystal data

**Table:**

[Cu(C_5_H_9_O_2_)_2_(C_6_H_7_NO)_2_]	*F*(000) = 510
*M**_r_* = 484.04	*D*_x_ = 1.360 Mg m^−^^3^
Monoclinic, *P*2_1_/*n*	Cu *K*α radiation, λ = 1.54180 Å
Hall symbol: -P 2yn	Cell parameters from 3855 reflections
*a* = 9.797 (5) Å	θ = 3.2–71.6°
*b* = 8.829 (5) Å	µ = 1.63 mm^−^^1^
*c* = 13.674 (5) Å	*T* = 150 K
β = 91.907 (5)°	Block, blue
*V* = 1182.1 (10) Å^3^	0.33 × 0.28 × 0.23 mm
*Z* = 2	

### Data collection

**Table:**

Oxford Super Nova diffractometer	2282 independent reflections
Radiation source: Micro-Focus (Cu) X-ray Source	2052 reflections with *I* > 2σ(*I*)
Graphite monochromator	*R*_int_ = 0.036
Detector resolution: 15.9948 pixels mm^-1^	θ_max_ = 71.8°, θ_min_ = 5.5°
ω/θ scans	*h* = −12→11
Absorption correction: multi-scan (*CrysAlis RED*; Oxford Diffraction, 2009)	*k* = −7→10
*T*_min_ = 0.615, *T*_max_ = 0.706	*l* = −16→16
6929 measured reflections	

### Refinement

**Table:**

Refinement on *F*^2^	Primary atom site location: structure-invariant direct methods
Least-squares matrix: full	Secondary atom site location: difference Fourier map
*R*[*F*^2^ > 2σ(*F*^2^)] = 0.040	Hydrogen site location: inferred from neighbouring sites
*wR*(*F*^2^) = 0.119	H atoms treated by a mixture of independent and constrained refinement
*S* = 1.06	*w* = 1/[σ^2^(*F*_o_^2^) + (0.0711*P*)^2^ + 0.5457*P*] where *P* = (*F*_o_^2^ + 2*F*_c_^2^)/3
2282 reflections	(Δ/σ)_max_ < 0.001
146 parameters	Δρ_max_ = 0.43 e Å^−^^3^
0 restraints	Δρ_min_ = −0.54 e Å^−^^3^

### Special details

**Table:**

Geometry. All e.s.d.'s (except the e.s.d. in the dihedral angle between two l.s. planes) are estimated using the full covariance matrix. The cell e.s.d.'s are taken into account individually in the estimation of e.s.d.'s in distances, angles and torsion angles; correlations between e.s.d.'s in cell parameters are only used when they are defined by crystal symmetry. An approximate (isotropic) treatment of cell e.s.d.'s is used for estimating e.s.d.'s involving l.s. planes.
Refinement. Refinement of *F*^2^ against ALL reflections. The weighted *R*-factor *wR* and goodness of fit *S* are based on *F*^2^, conventional *R*-factors *R* are based on *F*, with *F* set to zero for negative *F*^2^. The threshold expression of *F*^2^ > σ(*F*^2^) is used only for calculating *R*-factors(gt) *etc*. and is not relevant to the choice of reflections for refinement. *R*-factors based on *F*^2^ are statistically about twice as large as those based on *F*, and *R*-factors based on ALL data will be even larger.

### Fractional atomic coordinates and isotropic or equivalent isotropic displacement parameters (Å^2^)

**Table:**

	*x*	*y*	*z*	*U*_iso_*/*U*_eq_	
Cu1	0.5000	0.0000	0.5000	0.02121 (17)	
O1	0.52417 (16)	−0.22601 (17)	0.40801 (10)	0.0281 (3)	
O2	0.31138 (15)	−0.04928 (18)	0.54027 (11)	0.0284 (3)	
O3	0.28735 (16)	−0.28213 (18)	0.47823 (11)	0.0323 (4)	
N1	0.57203 (17)	−0.14564 (18)	0.59959 (12)	0.0231 (4)	
C1	0.5683 (2)	−0.1127 (2)	0.69555 (15)	0.0266 (4)	
H1	0.5305	−0.0185	0.7147	0.032*	
C2	0.6170 (2)	−0.2102 (3)	0.76689 (16)	0.0315 (5)	
H2	0.6142	−0.1836	0.8341	0.038*	
C3	0.6701 (2)	−0.3477 (3)	0.73850 (18)	0.0347 (5)	
H3	0.7055	−0.4168	0.7861	0.042*	
C4	0.6712 (2)	−0.3839 (3)	0.63980 (18)	0.0325 (5)	
H4	0.7049	−0.4792	0.6192	0.039*	
C5	0.6222 (2)	−0.2788 (2)	0.57143 (15)	0.0251 (4)	
C6	0.6241 (2)	−0.3092 (3)	0.46280 (17)	0.0323 (5)	
H6A	0.7156	−0.2834	0.4390	0.039*	
H6B	0.6090	−0.4186	0.4512	0.039*	
C7	0.2550 (2)	−0.1780 (2)	0.53540 (13)	0.0227 (4)	
C8	0.1446 (2)	−0.2117 (3)	0.60946 (15)	0.0280 (5)	
C9	0.2209 (3)	−0.2819 (4)	0.6974 (2)	0.0605 (9)	
H9A	0.2864	−0.2085	0.7251	0.091*	
H9B	0.2696	−0.3726	0.6765	0.091*	
H9C	0.1555	−0.3097	0.7470	0.091*	
C10	0.0713 (3)	−0.0693 (4)	0.6412 (2)	0.0593 (8)	
H10A	0.1382	0.0035	0.6680	0.089*	
H10B	0.0061	−0.0950	0.6914	0.089*	
H10C	0.0223	−0.0244	0.5847	0.089*	
C11	0.0425 (3)	−0.3258 (4)	0.5677 (2)	0.0659 (10)	
H11A	0.0909	−0.4175	0.5480	0.099*	
H11B	−0.0059	−0.2819	0.5105	0.099*	
H11C	−0.0233	−0.3516	0.6174	0.099*	
H101	0.434 (4)	−0.251 (4)	0.428 (2)	0.053 (9)*	

### Atomic displacement parameters (Å^2^)

**Table:**

	*U*^11^	*U*^22^	*U*^33^	*U*^12^	*U*^13^	*U*^23^
Cu1	0.0266 (3)	0.0185 (3)	0.0187 (3)	−0.00076 (14)	0.00306 (17)	0.00341 (14)
O1	0.0340 (8)	0.0301 (8)	0.0206 (7)	0.0000 (6)	0.0055 (6)	−0.0007 (6)
O2	0.0301 (8)	0.0233 (8)	0.0323 (8)	−0.0012 (6)	0.0069 (6)	0.0034 (6)
O3	0.0342 (8)	0.0389 (9)	0.0242 (7)	−0.0084 (6)	0.0074 (6)	−0.0125 (6)
N1	0.0272 (8)	0.0205 (8)	0.0219 (8)	−0.0017 (6)	0.0033 (6)	0.0019 (6)
C1	0.0305 (10)	0.0265 (10)	0.0229 (10)	−0.0038 (8)	0.0003 (8)	−0.0007 (8)
C2	0.0338 (11)	0.0375 (12)	0.0229 (10)	−0.0062 (9)	−0.0033 (8)	0.0053 (9)
C3	0.0305 (11)	0.0359 (12)	0.0371 (12)	−0.0027 (9)	−0.0050 (9)	0.0155 (10)
C4	0.0310 (11)	0.0247 (11)	0.0419 (13)	0.0032 (8)	0.0010 (9)	0.0074 (9)
C5	0.0255 (10)	0.0219 (10)	0.0282 (11)	−0.0009 (8)	0.0032 (8)	0.0024 (8)
C6	0.0370 (12)	0.0304 (11)	0.0298 (11)	0.0056 (9)	0.0068 (9)	−0.0018 (9)
C7	0.0260 (10)	0.0271 (10)	0.0148 (9)	−0.0013 (8)	−0.0011 (7)	0.0008 (7)
C8	0.0300 (11)	0.0317 (11)	0.0228 (10)	−0.0058 (8)	0.0069 (8)	−0.0031 (8)
C9	0.0534 (18)	0.096 (2)	0.0332 (14)	0.0017 (16)	0.0136 (12)	0.0251 (15)
C10	0.0640 (19)	0.0502 (18)	0.066 (2)	0.0039 (14)	0.0370 (16)	−0.0086 (15)
C11	0.0546 (18)	0.088 (2)	0.0568 (18)	−0.0427 (17)	0.0249 (15)	−0.0278 (17)

### Geometric parameters (Å, º)

**Table:**

Cu1—N1	1.9855 (17)	C4—H4	0.9500
Cu1—N1^i^	1.9855 (17)	C5—C6	1.510 (3)
Cu1—O2	1.9937 (17)	C6—H6A	0.9900
Cu1—O2^i^	1.9937 (17)	C6—H6B	0.9900
Cu1—O1^i^	2.3748 (18)	C7—O3	1.254 (3)
Cu1—O1	2.3748 (18)	C7—C8	1.535 (3)
O1—C6	1.418 (3)	C8—C11	1.518 (3)
O1—H101	0.95 (4)	C8—C10	1.518 (4)
O2—C7	1.265 (3)	C8—C9	1.526 (4)
O3—C7	1.254 (3)	C9—H9A	0.9800
N1—C5	1.336 (3)	C9—H9B	0.9800
N1—C1	1.346 (3)	C9—H9C	0.9800
C1—C2	1.375 (3)	C10—H10A	0.9800
C1—H1	0.9500	C10—H10B	0.9800
C2—C3	1.382 (4)	C10—H10C	0.9800
C2—H2	0.9500	C11—H11A	0.9800
C3—C4	1.387 (4)	C11—H11B	0.9800
C3—H3	0.9500	C11—H11C	0.9800
C4—C5	1.391 (3)		
			
N1—Cu1—N1^i^	180.00 (7)	C4—C5—C6	121.8 (2)
N1—Cu1—O2	88.90 (7)	O1—C6—C5	113.40 (18)
N1^i^—Cu1—O2	91.10 (7)	O1—C6—H6A	108.9
N1—Cu1—O2^i^	91.10 (7)	C5—C6—H6A	108.9
N1^i^—Cu1—O2^i^	88.90 (7)	O1—C6—H6B	108.9
O2—Cu1—O2^i^	180.0	C5—C6—H6B	108.9
N1—Cu1—O1^i^	102.73 (7)	H6A—C6—H6B	107.7
N1^i^—Cu1—O1^i^	77.27 (7)	O3—C7—O2	124.95 (19)
O2—Cu1—O1^i^	85.84 (6)	O3—C7—O2	124.95 (19)
O2^i^—Cu1—O1^i^	94.16 (6)	O3—C7—C8	117.88 (18)
N1—Cu1—O1	77.27 (7)	O3—C7—C8	117.88 (18)
N1^i^—Cu1—O1	102.73 (7)	O2—C7—C8	117.07 (17)
O2—Cu1—O1	94.16 (6)	C11—C8—C10	110.2 (2)
O2^i^—Cu1—O1	85.84 (6)	C11—C8—C9	109.0 (3)
O1^i^—Cu1—O1	180.0	C10—C8—C9	109.6 (2)
C6—O1—Cu1	103.56 (12)	C11—C8—C7	110.48 (18)
C6—O1—H101	111 (2)	C10—C8—C7	112.25 (19)
Cu1—O1—H101	86.1 (19)	C9—C8—C7	105.14 (19)
C7—O2—Cu1	126.06 (13)	C8—C9—H9A	109.5
C5—N1—C1	119.59 (18)	C8—C9—H9B	109.5
C5—N1—Cu1	119.87 (14)	H9A—C9—H9B	109.5
C1—N1—Cu1	120.52 (14)	C8—C9—H9C	109.5
N1—C1—C2	122.4 (2)	H9A—C9—H9C	109.5
N1—C1—H1	118.8	H9B—C9—H9C	109.5
C2—C1—H1	118.8	C8—C10—H10A	109.5
C1—C2—C3	118.4 (2)	C8—C10—H10B	109.5
C1—C2—H2	120.8	H10A—C10—H10B	109.5
C3—C2—H2	120.8	C8—C10—H10C	109.5
C2—C3—C4	119.4 (2)	H10A—C10—H10C	109.5
C2—C3—H3	120.3	H10B—C10—H10C	109.5
C4—C3—H3	120.3	C8—C11—H11A	109.5
C3—C4—C5	119.1 (2)	C8—C11—H11B	109.5
C3—C4—H4	120.4	H11A—C11—H11B	109.5
C5—C4—H4	120.4	C8—C11—H11C	109.5
N1—C5—C4	121.0 (2)	H11A—C11—H11C	109.5
N1—C5—C6	117.14 (18)	H11B—C11—H11C	109.5
			
N1—Cu1—O1—C6	−23.50 (13)	Cu1—N1—C5—C4	−178.92 (16)
N1^i^—Cu1—O1—C6	156.50 (13)	C1—N1—C5—C6	−179.83 (19)
O2—Cu1—O1—C6	−111.43 (13)	Cu1—N1—C5—C6	1.5 (2)
O2^i^—Cu1—O1—C6	68.57 (13)	C3—C4—C5—N1	−1.3 (3)
N1—Cu1—O2—C7	−61.13 (16)	C3—C4—C5—C6	178.2 (2)
N1^i^—Cu1—O2—C7	118.87 (16)	Cu1—O1—C6—C5	30.6 (2)
O1^i^—Cu1—O2—C7	−163.98 (16)	N1—C5—C6—O1	−25.3 (3)
O1—Cu1—O2—C7	16.02 (16)	C4—C5—C6—O1	155.2 (2)
O2—Cu1—N1—C5	106.78 (16)	O3—O3—C7—O2	0.00 (19)
O2^i^—Cu1—N1—C5	−73.22 (16)	O3—O3—C7—C8	0.00 (10)
O1^i^—Cu1—N1—C5	−167.74 (15)	Cu1—O2—C7—O3	−24.1 (3)
O1—Cu1—N1—C5	12.26 (15)	Cu1—O2—C7—O3	−24.1 (3)
O2—Cu1—N1—C1	−71.87 (16)	Cu1—O2—C7—C8	152.14 (14)
O2^i^—Cu1—N1—C1	108.13 (16)	O3—C7—C8—C11	−31.3 (3)
O1^i^—Cu1—N1—C1	13.62 (16)	O3—C7—C8—C11	−31.3 (3)
O1—Cu1—N1—C1	−166.38 (16)	O2—C7—C8—C11	152.3 (2)
C5—N1—C1—C2	1.4 (3)	O3—C7—C8—C10	−154.7 (2)
Cu1—N1—C1—C2	−179.99 (16)	O3—C7—C8—C10	−154.7 (2)
N1—C1—C2—C3	−0.8 (3)	O2—C7—C8—C10	28.8 (3)
C1—C2—C3—C4	−0.8 (3)	O3—C7—C8—C9	86.3 (2)
C2—C3—C4—C5	1.8 (3)	O3—C7—C8—C9	86.3 (2)
C1—N1—C5—C4	−0.3 (3)	O2—C7—C8—C9	−90.2 (3)

Symmetry code: (i) −*x*+1, −*y*, −*z*+1.

### Hydrogen-bond geometry (Å, º)

**Table:**

*D*—H···*A*	*D*—H	H···*A*	*D*···*A*	*D*—H···*A*
O1—H101···O3	0.95 (4)	1.64 (4)	2.588 (2)	171 (3)
C2—H2···O3^ii^	0.95	2.57	3.289 (3)	132
C4—H4···O3^iii^	0.95	2.50	3.392 (3)	157

Symmetry codes: (ii) *x*+1/2, −*y*−1/2, *z*+1/2; (iii) −*x*+1, −*y*−1, −*z*+1.
